# Vitamin D inhibits TNF-α induced apoptosis of human nucleus pulposus cells through regulation of NF-kB signaling pathway

**DOI:** 10.1186/s13018-021-02545-9

**Published:** 2021-06-28

**Authors:** Cun Zhang, Tong Tong, De-chao Miao, Lin-feng Wang

**Affiliations:** Department of Spine Surgery, The Third Hospital of HeBei Medical University, 139 Ziqiang Road, Shijiazhuang, 050051 China

**Keywords:** Vitamin D, TNF-α, Nucleus pulposus cells, Apoptosis, NF-κB pathway

## Abstract

**Background:**

To observe the effects of vitamin D on the apoptotic human nucleus pulposus cells under tumor necrosis factor-α (TNF-α) treatment.

**Methods:**

The gene expression data was downloaded from the NCBI Gene Expression Omnibus (GEO) database (https://www.ncbi.nlm.nih.gov/geo/query/acc.cgi?acc=GSE34095). Differentially expressed genes between degenerative disc and non-degenerative disc were performed by R software. Gene ontology (GO) and Kyoto Encyclopedia of Genes and Genome (KEGG) pathway enrichment analyses were performed using The Database for Annotation, Visualization and Integrated Discovery (DAVID). Then, the human nucleus pulposus tissue was harvested from 12 patients according to the modified Pfirrmann classification and human nucleus pulposus cells were obtained from digestion of herniated nucleus pulposus tissue. The collected nucleus pulposus cells were treated with different concentration of TNF-α, and cellular apoptosis was measured by flow cytometry. Then, human nucleus pulposus cells were divided into following groups: normal culture medium, TNF-α treated, TNF-α, and vitamin D-treated groups. Cellular apoptosis rate was quantified by flow cytometry. Protein expression of p-p65, p65, and IkBa was detected with western blot analysis.

**Results:**

A total of 536 differentially expressed genes were identified through bioinformatic analysis. KEGG pathway revealed that NF-kB signaling pathway was involved in the process of disc degeneration. In the NP cell cultures, vitamin D significantly increased cell proliferation potency. Furthermore, vitamin D inhibited TNF-α induced apoptosis of human nucleus pulposus cells. Vitamin D reduced the phospho-NF-κB/p65 expression in the TNF-α-treated NP cells.

**Conclusion:**

Vitamin D can attenuate TNF-α-induced NP cells apoptosis through interfering with the NF-κB pathway.

## Background

Low back pain is a common disease in orthopedics clinic [1]. It is estimated that 80% of adults will have a lower back pain in their lives [2]. With the advent of an aging society, low back pain has become a common and frequently occurring [3]. Low back pain seriously endangers the health of the population, which also has an important impact on social and economic progress [4, 5]. Lumbar disc degeneration has been recognized as an important risk factor for low back pain [6]. The human intervertebral disc is mainly composed of the annulus fibrosus and the nucleus pulposus. Especially, nucleus pulposus cell apoptosis caused by various factors is the major cause of lumbar disc degeneration [7].

At the molecular level, cytokines such as IL-1β or TNF-α were related to the degeneration of the intervertebral disc [8]. TNF-α significantly causes excessive apoptosis of nucleus pulposus cells. Therefore, the control of apoptosis is of great significance for the treatment of TNF-α caused nucleus pulposus cell apoptosis [9, 10].

Vitamin D is a steroid hormone, which mainly used to maintain the stability of serum calcium and phosphorus concentration [11]. Recently, studies reported that vitamin D has the role of regulating collagen matrix in the human bone [12]. Vitamin D receptor gene is associated with the response to anti-osteoporotic therapy in postmenopausal women from southern Italy [13]. Moreover, vitamin D receptor polymorphisms may be associated with abnormal acetabular morphology leading to dysplasia of the hip [14].

Vitamin D receptors were found in various tissues and organs. Vitamin D can directly or indirectly regulate more than 200 genes, and its effects include promoting cell differentiation, inhibiting cell apoptosis, and inhibiting angiogenesis and anti-inflammatory [15].

Vitamin D plays a key role in mineral balance in the body and bone health [16]. The lack of vitamin D is a universal problem in the world, especially in countries and regions in the northern hemisphere [17]. Foccillo et al. [18] revealed that the activation of the inflammatory cascade may induce reduction of vitamin D levels, and fall is associated with impaired bone health.

Videman et al. [19] found that the vitamin D receptor expression level was related to the degeneration of the intervertebral disc.

However, whether vitamin D inhibited the TNF-α induced nucleus pulposus cells apoptosis was unknown. Moreover, the mechanism of vitamin D inhibited the TNF-α induced nucleus pulposus cells apoptosis was still need to be further explored.

## Material and methods

### Bioinformatic analysis of GSE34095

Affymetrix microarray data representing three human degenerative and three human non-degenerative intervertebral discs tissues were downloaded from the Gene Expression Omibus repository (www.ncbi.nih.gov/geo) under accession number GSE34095. Raw data were normalized by the variance stabilization normalization method by R software (http://www.r-project.org/). Differentially expressed genes were analyzed by the linear models for microarray data (Limma) package in R software [20]. The heatmaps and volcano plot were generated using R software with pheatmap package and volcano plots [21].

To explore the biological implication of the differentially expressed genes, we performed gene-term enrichment analysis using Gene Ontology (GO) categories and Kyoto Encyclopedia of Genes and Genomes (KEGG) pathway analysis by using the DAVID Functional Annotation Chart tool [22]. GO terms including biological process (BP), cellular component (CC), and molecular function (MF).

### Cell culture and treatments

Human nucleus pulposus cells were isolated from normal intervertebral disc tissues as described previously [23]. In brief, human nucleus pulposus tissue was separated by an experienced surgeon. Human nucleus pulposus tissue was sheared into starch paste by asepsis scissors and then digested with collagenase-II (0.5%) for 2 h at 37°C. After filtration by aseptic cell sieves, the cells were washed and collected by centrifugation (100 g, 2 min, three times) with PBS. Cells were then cultured in Dulbecco’s modified Eagle’s medium (DMEM, Gibco, Gaithersburg, MD, USA) supplemented with 10% fetal bovine serum (FBS, Gibco, Gaithersburg, MD, USA) at 37 °C and 5% CO_2_.

### Human nucleus pulposus cells treated in TNF-α condition

Human nucleus pulposus cells were treated with TNF-α to mimic the injury model. Human nucleus pulposus cells were cultured in different concentration of TNF-α to identify the optimal dose of TNF-α. In brief, human nucleus pulposus cells were cultured in 6-well plates, and the medium was replaced by 1 ml of basic medium mixed with different concentration of TNF-α after the cell confluence attained 80%. Then, human nucleus pulposus cells were harvested for further analysis. To further identify the role of vitamin D on the apoptosis of nucleus pulposus cells, nucleus pulposus cells were pretreated with vitamin D for 6 h and then incubated with TNF-α.

### Cell count kit-8 (CCK-8) assay

CCK-8 assay (Solarbio, Beijing, China) was used for the measurement of cell viability. Briefly, human nucleus pulposus cells were digested and then seeded in 96-well plates (5 × 10^3^/well) for 12 h; after that, the cells were treated with vitamin D and then subjected to TNF-α treatment for indicated times. Then, the cells in each well were incubated with CCK-8 solution for 2 h at 37 °C. Then, the absorbance was checked with an automatic microplate reader at 450 nm (ELX800, BioTek Instrument, Inc., USA).

### Flow detection of apoptosis

To analyze the effect of vitamin D on apoptosis, we determined Annexin V staining by flow cytometric analyses. In brief, nucleus pulposus cells were incubated in 6-well plates and divided into following groups: control group, TNF-α-treated group, and vitamin D + TNF-α-treated group. Nucleus pulposus cells were then underwent routine digestion and centrifugation. Nucleus pulposus cells were incubated with PI and Annexin V staining at dark for 1 h and then washed with PBS for three times. FACSAriaTM III (BD Biosciences, USA) was used to detect cell apoptosis among the three groups. Total apoptotic rate = early apoptotic rate (Q3) + late apoptotic rate (Q2).

### Quantitative real-time PCR (qPCR)

To perform qRT-PCR, RNA was isolated using Trizol (Invitrogen) from nucleus pulposus cells. From 1 mug of total RNA, cDNA synthesis was performed with a reverse transcriptase kit (TaKaRa, Shiga, Japan) according to the manufacturer’s instructions. For RT-PCR, Bcl-2 and cleaved caspase-3 primers were used. The relative quantity of Bcl-2 and cleaved caspase-3 was normalized to GAPDH. The expression of Bcl-2 and cleaved caspase-3 was detected using qPCR with SYBR Green Mix Kits (Applied Biosystems). All results were quantitated using the 2^-ΔΔCt^ relative quantification method.

### Western blot detection

At the end of incubation, proteins were extracted from cells and quantified using a BCA protein assay kit. For immunoblotting, samples (20–30 μg/well) were separated by electrophoresis, transferred into polyvinylidene difluoride (PVDF) membranes (Merck Millipore, Billerica, MA, USA), and then blocked with 5% non-fat milk at room temperature 1.5 h. The primary antibodies were incubated with different membranes overnight at 4°C. The secondary antibodies (horseradish peroxidase-conjugated goat anti-rabbit or goat anti-mouse) were incubated with the membranes for 1.5 h. The immunoreactive proteins on the PVDF membranes were visualized using a 5200 Tanon electro chemiluminescence (ECL) detection system (Shanghai, China).

### Statistical analysis

All experiments were executed independently repeated at least three times. All results were demonstrated as means ± S.D. Student’s t test and one-way analysis of variance (ANOVA) analysis were used to determine the significance differences between two experimental groups with statistic software SPSS 20.0. The values of P < 0.05 were considered statistically significant.

## Results

### Differentially expressed genes between degenerative and non-degenerative intervertebral discs tissues

**Fig. 1:**
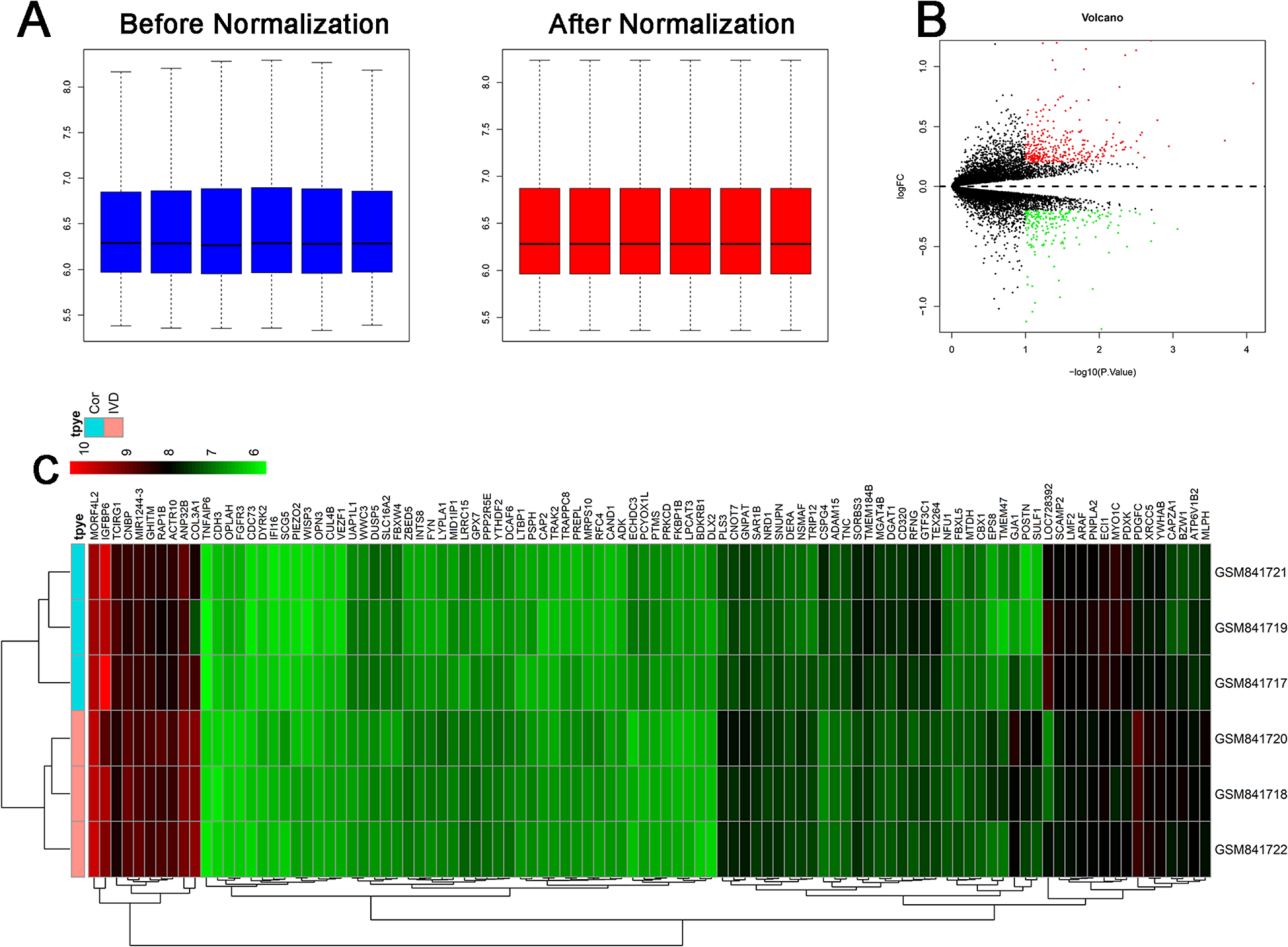
Bioinformatic analysis of GSE34095. **A** Expression level of GSE34095 before and after normalization. **B** Volcano plot of the differentially expressed genes between degenerative and non-degenerative intervertebral discs tissues. **C** Heatmap of the top 100 differentially expressed genes between degenerative and non-degenerative intervertebral discs tissues

After normalization of the raw data, the average expression data were identical and then used for further comparative analysis of mRNA expression level (Fig. 1A). A total of 539 differentially expressed genes were identified based on the criteria of P <0.05 and log_2_ fold change > 1, including 336 upregulated genes and 203 downregulated genes. Volcano plot of the differentially expressed genes between degenerative and non-degenerative intervertebral discs tissues can be seen in Fig. 1B. Heatmap of top 100 most differentially expressed genes that define the clusters depicted in Fig. 1C.

**Fig. 2:**
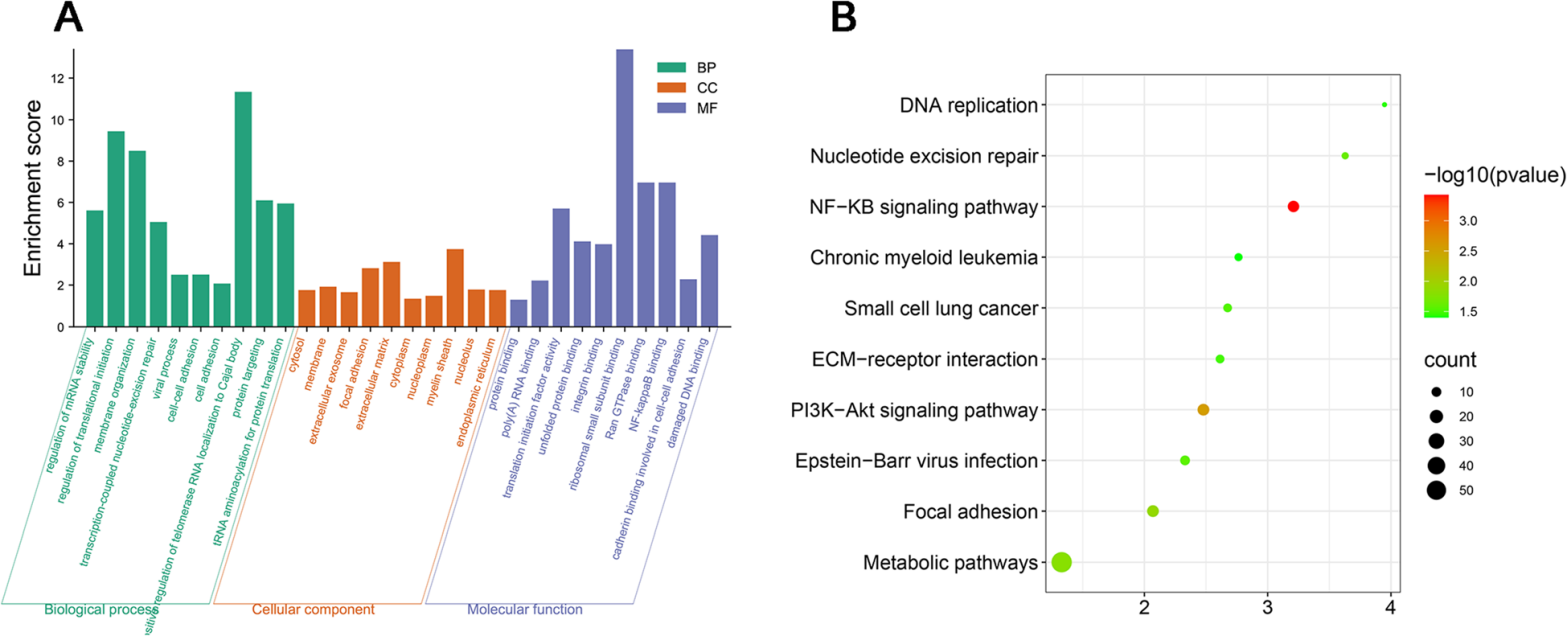
Gene ontology and KEGG pathway analysis of the differentially expressed genes. **A** Gene ontology (biological process, cellular component and molecular function) of the differentially expressed genes. **B** KEGG pathway analysis of the differentially expressed genes

Moreover, biological process ontology contains mainly including regulation of mRNA stability, regulation of translational initiation, membrane organization, transcription-coupled nucleotide-excision repair, viral process, cell–cell adhesion, cell adhesion, positive regulation of telomerase RNA localization to Cajal body, protein targeting, and tRNA aminoacylation for protein translation (Fig. 2A).

Cellular component contains mainly including cytosol, membrane, extracellular exosome, focal adhesion, extracellular matrix, cytoplasm, nucleoplasm, myelin sheath, nucleolus, and endoplasmic reticulum (Fig. 2A).

Molecular function contains mainly including protein binding, poly(A) RNA binding, translation initiation factor activity, unfolded protein binding, integrin binding, ribosomal small subunit binding, Ran GTPase binding, NF-kappaB binding, cadherin binding involved in cell–cell adhesion, and damaged DNA binding (Fig. 2A).

KEGG pathway mainly including NF-KB signaling pathway, PI3K-Akt signaling pathway, focal adhesion, metabolic pathways, nucleotide excision repair, epstein-Barr virus infection, small cell lung cancer, ECM-receptor interaction, DNA replication, and chronic myeloid leukemia (Fig. 2B).

### Identification of nucleus pulposus cells

**Fig. 3:**
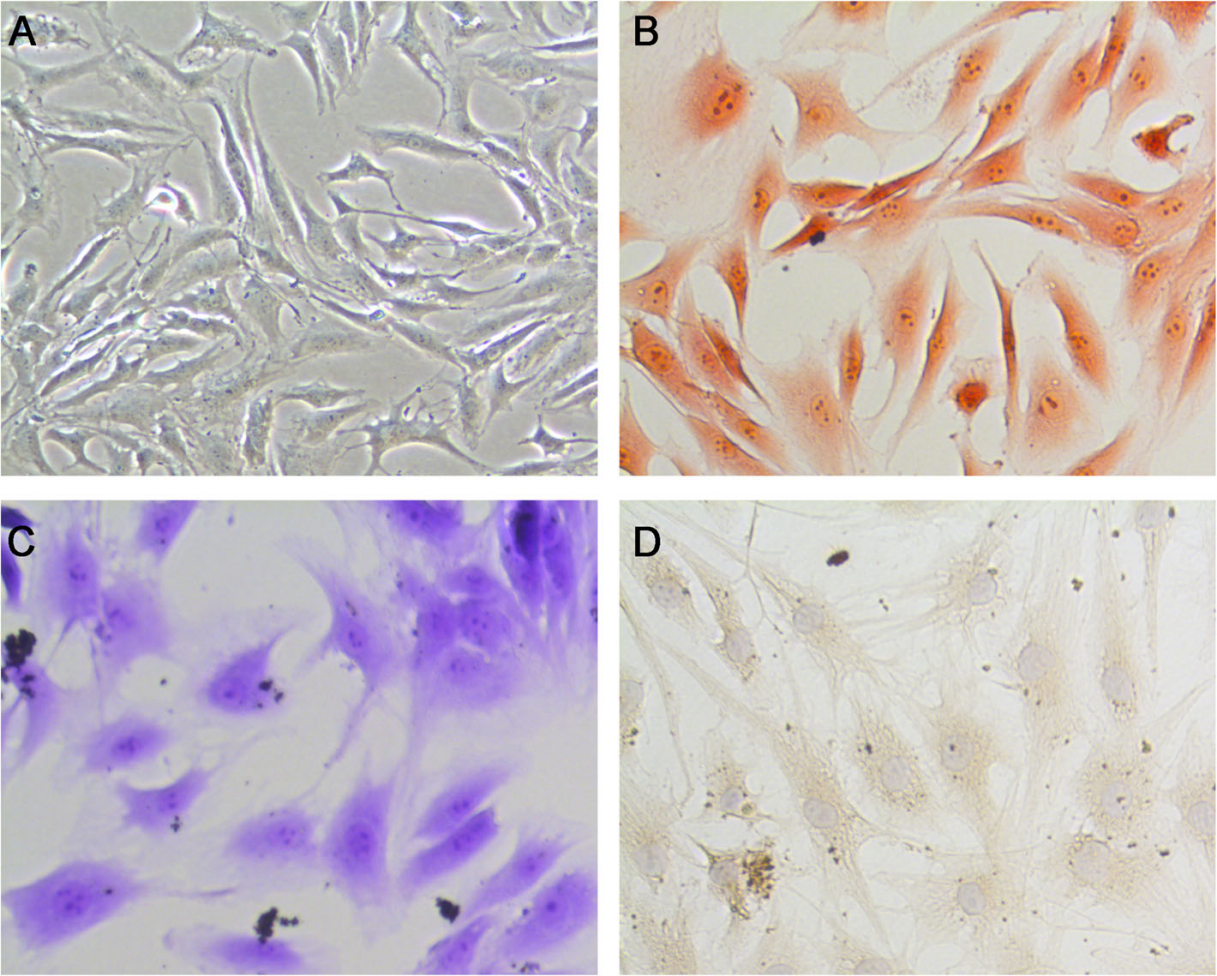
Identification of nucleus pulposus cells. **A** The gross morphology of the nucleus pulposus cells was observed. **B** Safranin O staining of nucleus pulposus cells. **C** Toluidine blue staining of nucleus pulposus cells. **D** Immunohistochemically for collagen II of nucleus pulposus cells

Nucleus pulposus cells are long and spindle-shaped in appearance (Fig. 3A). Nucleus pulposus cells are Safranin O staining positive (Fig. 3B). Moreover, nucleus pulposus cells are Toluidine blue staining positive (Fig. 3C) and immunohistochemically for collagen II positive (Fig. 3D). Those nucleus pulposus cells were more chondrocyte-like cells.

**Fig. 4:**
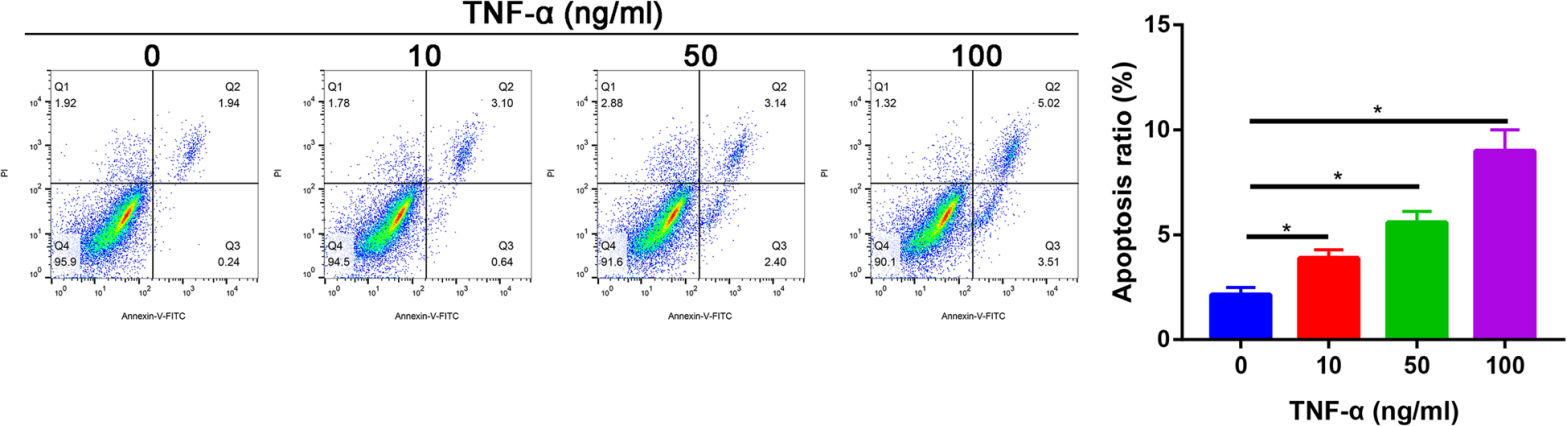
The apoptotic ratio of nucleus pulposus cells in different concentration of TNF-α groups

**Fig. 5:**
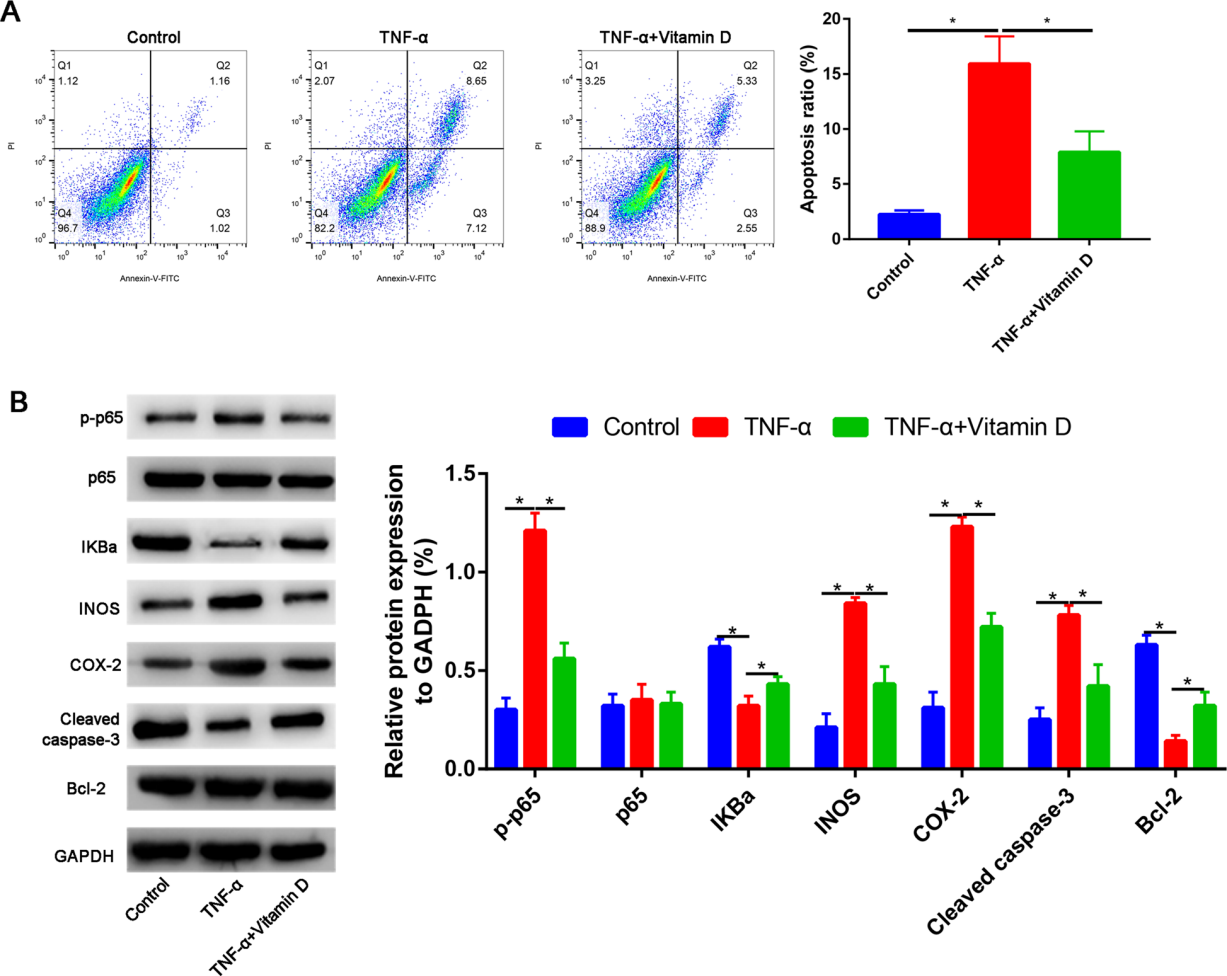
**A** Vitamin D partially reversed the TNF-α induced the apoptosis of nucleus pulposus cells. **B** Western blot assay to assess the Bcl-2, cleaved caspase-3, p-p65, p-65, IKBa, INOS, and COX-2 expressions

**Fig. 6:**
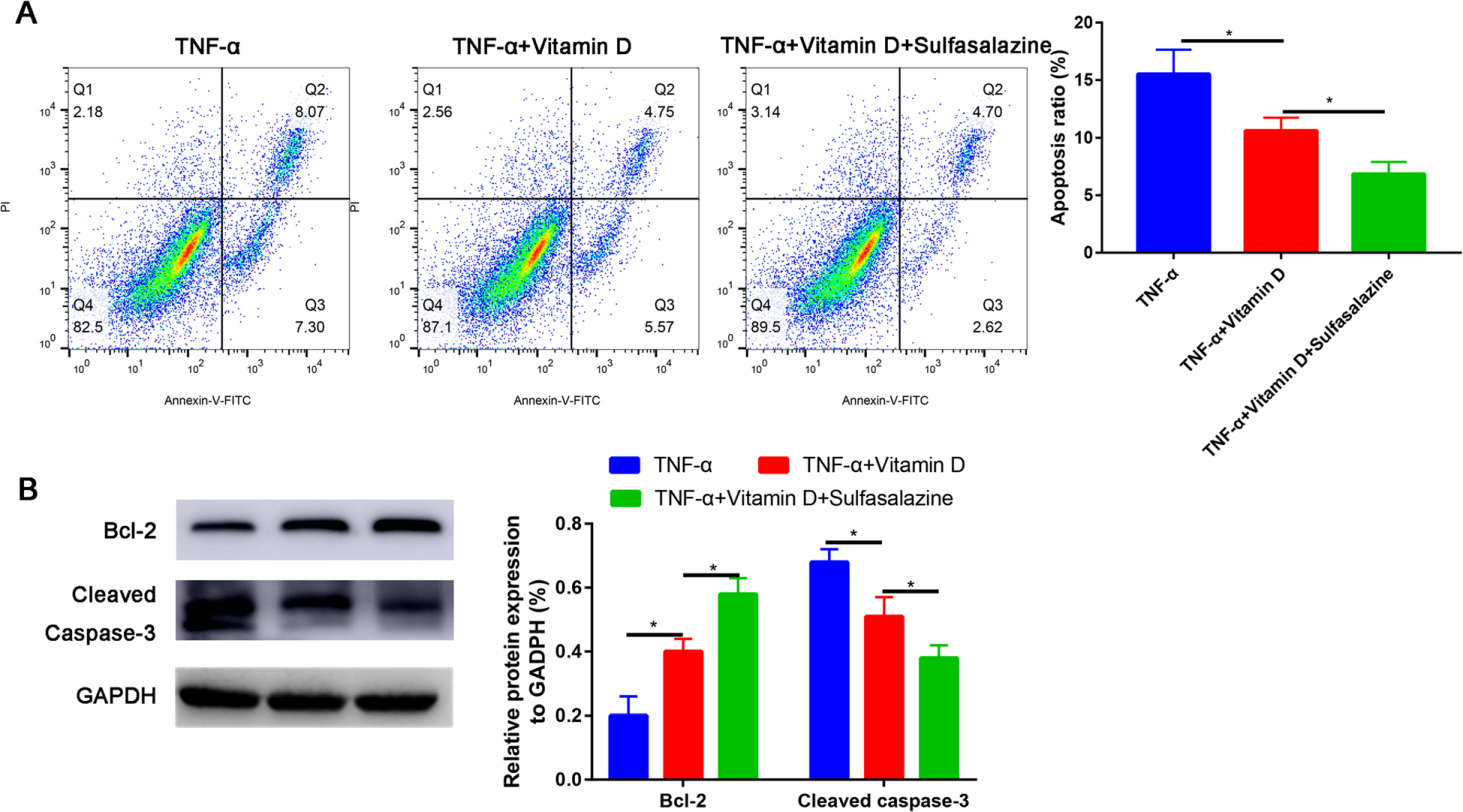
Sulfasalazine partially recovered the apoptosis rate of TNF-α-treated nucleus pulposus cells. **A** Cell apoptosis was determined by flow cytometry assay. **B** Western blot assay to assess the Bcl-2 and cleaved caspase-3 expression

### TNF-α treatment significantly increased the apoptosis in a dose-response manner

Compared with the control group, the apoptosis of nucleus pulposus cells increased in TNF-α-treated group in a dose-dependent manner. TNF-α, 100 ng/ml, caused extensive apoptosis (apoptotic index, 19.6%±4.6%) than other doses (10 and 30 ng/ml) (Fig. 4).

### Vitamin D partially reversed the TNF-α-induced nucleus pulposus cells apoptosis

Consistent with previous results, TNF-α treatment significantly increased the apoptosis of nucleus pulposus cells. However, pretreatment with vitamin D partially reversed the TNF-α-induced nucleus pulposus cells apoptosis, and the difference is statistically significant (P<0.05, Fig. 5 A). The expressions of apoptotic-related markers were detected by Western blot. Results found that the cleaved caspase-3 expression was upregulated, while Bcl-2 expression was downregulated in the TNF-α group than in the control group. Compared with the TNF-α group, the cleaved caspase-3 expression was partially downregulated, while Bcl-2 expression was upregulated (Fig. 5B).

### Vitamin D partially reversed the TNF-α-induced nucleus pulposus cells apoptosis through NF-kB signaling pathway

We firstly measured the p-p65, p65, and IkBa protein expressions in control, TNF-α, and TNF-α + vitamin D groups (Fig. 5B). Compared with the control group, TNF-α significantly increased the p-p65 expression, while this effect was partially blocked by the vitamin D (Fig. 5B). However, there was no significant difference between the p65 expression in in control, TNF-α, and TNF-α + vitamin D groups (Fig. 5B).

To further test whether NF-kB signaling pathway functions in TNF-α-induced nucleus pulposus cells apoptosis, sulfasalazine was used to inhibit NF-kB pathway. We found that NF-kB signaling pathway inhibitor (sulfasalazine) could partially recovered the apoptosis rate (Fig. 6A). Western blot was then performed to validate the result of flow cytology (Fig. 6B).

## Discussion

This is the first study that revealed that vitamin D significantly downregulated TNF-α induced nucleus pulposus cells apoptosis through NF-kB signaling pathway. Firstly, we performed bioinformatic analysis between degenerative and non-degenerative intervertebral discs tissues using GSE34095. A total of 539 differentially expressed genes were identified and these differentially expressed genes mainly enriched in the NF-kB signaling pathway. These results suggested that NF-kB signaling pathway participated into the lumbar disc degeneration. Then, we establish the nucleus pulposus cells injury model and identified the optimal dose of TNF-α.

Flow cytometry results proved that vitamin D significantly suppressed TNF-α induced apoptosis of nucleus pulposus cells. We further showed that vitamin D exerts anti-apoptosis effect by inhibiting the NF-kB signaling pathway. The lumbar disc degeneration is influenced by both the genetic and environmental factors. Vitamin D affects collagen and proteoglycans synthesis. Moreover, vitamin D play an important role in regulating the differentiation, proliferation and formation of chondrocytes. These previous studies prompted us that vitamin D may be an alternative for treatment of lumbar disc degeneration.

Which signaling pathway affects the degeneration of the intervertebral disc is still unknown. Thus, we firstly performed bioinformatic analysis about degenerative and non-degenerative intervertebral discs tissues using GSE34095 profile. We selected NF-kB signaling pathway for further study. Zhu et al. [24] found that NF-kB signaling pathway is involved in rat model of lumbar disc herniation. In vitro experiments have confirmed that vitamin D has regulated effect on chondrogenic differentiation at all stages [25].

The signaling pathway involving the transcription factor NF-kB is considered a typical pro-inflammatory pathway. We evaluated the regulatory role of vitamin D in regulating the NF-kB signaling pathway. We found that TNF-α significantly activated the NF-kB signaling pathway, while the activated state of NF-kB signaling pathway was partially blocked by vitamin D.

The limitations of the study cannot be ignored. Firstly, in vivo animal experiment was lack. Secondly, vitamin D via which receptor exerts its protection effects was unknown. The oral dose of vitamin D needs for further studies to verify.

Based on these presented findings, we draw the conclusion that the NF-kB signaling pathway participated into the lumbar disc degeneration. Vitamin D partly attenuates TNF-α-induced apoptosis of human nucleus pulposus cells through regulating the NF-κB pathway.

## Conclusion

This study sheds a light on the protective role of vitamin D on the prevention of lumbar disc degeneration and finally contributes to understanding the mechanism underlying the positive effects of vitamin D supplementation on intervertebral discs.

## Data Availability

All the data pertaining to the present study have been included in figure in the manuscript, and the authors are willing to share the raw data upon reasonable request.

## Abbreviations

TNF-α: Tumor necrosis factor-α
GEO: Gene Expression Omnibus
GO: Gene ontology
KEGG: Kyoto Encyclopedia of Genes and Genomes
DAVID: The Database for Annotation, Visualization and Integrated Discovery
BP: Biological process
CC: Cellular component
MF: Molecular function
CCK-8: Cell count kit-8
PVDF: Polyvinylidene difluoride
ECL: Electrochemiluminescence
ANOVA: One-way analysis of variance
